# What Matters Most to Older Chinese Adults

**DOI:** 10.1177/10436596211053655

**Published:** 2021-10-26

**Authors:** Liza Lai Shan Choi, Piera Jung, Marti Harder, Kelly Zhang

**Affiliations:** 1 Mount Royal University, Calgary, Alberta, Canada; 2 Vancouver Island University, Nanaimo, British Columbia, Canada

**Keywords:** older Chinese adults, voices, what matters most

## Abstract

**Introduction:**

Although an abundance of gerontological research has focused on subjective well-being, quality of life, and life satisfaction, we know little about what matters most to older adults in sub-cultural groups. The purpose of this study was to explore what matters to older Chinese adults.

**Methods:**

The study used a qualitative interpretive design, drawing influences from phenomenology and constructed meaning through participants’ lived experiences.

**Results:**

After data analysis, a core theme of *cultural foundations* and categorical themes emerged. This study emphasized the importance of hearing the voices of Chinese older adults and how they viewed well-being, quality of life, life satisfaction, and health care.

**Discussion:**

The findings of this study have added to the body of existing knowledge of what matters most to older adults. These insights may advance nursing as it pertains to culturally congruent health care.

## Introduction

### Problem Formulation

Challenges faced by older Chinese adults as newcomers or immigrants to North America, specifically Canada, are well documented (Chow, 2012; Koehn et al., 2018; S. Liu et al., 2020; Zhu & Zhang, 2019). Contributing factors include communications barriers, loneliness, and insufficient access to social services (J. Li et al., 2018). If unaddressed, these issues reduce resilience and ability to cope with health issues that may accompany aging (Kung, 2004; Lai & Chau, 2007; Pang et al., 2003; B. Wu et al., 2010). Using interpretive description and phenomenology methods, we explored the lived experiences of older Chinese adults over the age of 70, in two Western Canadian cities. The study gives a voice to the participants, through their stories and perspectives on their aging journeys (Polit & Beck, 2017). Thematic data analysis is undertaken to describe what matters most to the older adults. Drawing on their perspectives contributes to the nursing profession in relation to culturally congruent health care for transcultural groups.

### Purpose

The main purpose of this study is to explore what matters to Chinese older adults, particularly in relation to subjective well-being, quality of life, and life satisfaction. The objectives of this study were (a) to determine what Chinese older adults value in their lives; (b) to document and understand their lived experiences through individual interviews; (c) to examine their Canadian living experience as newcomers and/or immigrants; and (d) to delineate the cultural needs of Chinese older adults. The participants’ voices in this study will help to illuminate the everyday strengths and challenges of growing old in areas where the culture is different from their country of origin.

## Literature Review

Canada’s population is projected to become significantly more ethnically and culturally diverse by 2031 (Statistics Canada, 2018a). Chinese older adults were chosen for this study as they form a growing sector of the Western Canadian population. The age of immigration, level of education, and fluency in Canada’s two official languages (English and French) contribute to increased reliance on public funds, higher rates of social isolation, and increased mortality (Government of Canada, 2005, 2021). Statistics Canada (2018b) reports 12% of British Columbia residents and 5% of Alberta residents speak Chinese (either Mandarin or Cantonese). As health care providers, we strive to promote physical, mental, and social healthy outcomes for immigrants living in the community (Chow, 2010; Koehn et al., 2018; Tieu & Konnert, 2015; Zhu & Zhang, 2019). In a review of immigrants’ access to health care, Wang et al. (2019) cited minimal focus specifically on older adults, the most vulnerable among the immigrant population. The process of shifting customs, values, and behaviors to another culture due to relocation is broadly defined as acculturation (Abraido-Lanza et al., 2006; S. Liu et al., 2020; Mao et al., 2020; Serafica et al., 2019; Tieu & Konnert, 2015). In addition to a steep learning curve about Canadian cultural norms, Chinese older adults’ traditional values from their country of origin were disrupted, causing identity conflict because of a constant need to navigate between two cultures while adapting to new environments (J. Li et al., 2018; S. Liu et al., 2020; Teh et al., 2020; Ward, 2013). The lived experiences of immigrants must be considered in relation to the process of acculturation because of the diverse differences in cultures (Ward, 2013).

Chinese immigrants’ transition to Canada has disrupted their traditional notions of social support from family, friends, and society (Gao et al., 2021; M. Li et al., 2021; J. Liu et al., 2020). Leaving their existing social networks behind, many older adults immigrate to Canada with their adult children to provide support to their children’s growing families (M. Li et al., 2021). Older adults’ traditional cultural values of family cohesiveness and filial responsibility are deeply rooted, but are challenged by individualism and the favored independent living in Western society, requiring them to modify their expectations (Chiang-Hanisko, 2010; Liou & Shenk, 2016; J. Liu et al., 2020). Lou and Ng (2012) described that a relationship-focused and family-oriented approach is essential for building and maintaining resilience among Chinese older adults, especially when cultural and traditional expectations of “old age” are not actualized.

Recent studies have focused on capacity-building for older adults to aid their sense of purpose in life, increasing their ability to positively cope with health issues (Irving et al., 2017; MacLeod et al., 2016; Stones & Gullifer, 2016). Gao et al. (2021) confirmed that older Chinese immigrants experienced higher levels of functional limitations than their counterparts in their country of origin, due to barriers related to language and transportation, which led to feelings of isolation, and a lack of independence and social support. Immigrants have greater loneliness than those born in Canada, supporting the notion that immigrant status is related to late-life social isolation (Z. Wu & Penning, 2015). Late-life isolation leads not only to mental disease, it also contributes to decreased health literacy because Chinese older adults are more likely to receive health-related information only through discussions with people that they trust (King-Shier et al., 2018; Zhang et al., 2020). Social support allows Chinese older adults to be better informed with culturally sensitive and linguistically appropriate health care information, adding to their resilience and ability to cope with aging-related changes (Chow, 2010; Luo & Menec, 2018). The day-to-day challenges that Chinese older adults face are well documented. To date, there are few reviews that explore the complexity of traditional meanings and interpretations that underpin life priorities and health decisions made by Chinese older adults living in Canada.

## Method

### Design

The study uses a qualitative interpretive design, with influences from phenomenology, constructing meaning through participants’ lived experiences. Four main components of the research design are: theoretical scaffolding through evolving literature reviews, sample selection criteria, data collection through interviews, and data interpretation using a phenomenological approach. Phenomenology provides a meaning-oriented image that serves to help people reflect on their experiences and encourages a deeper understanding of these experiences (Matua, 2015). To gain understanding through the lens of the participants, qualitative interviews and subsequent analysis of the interviews and participatory observations illuminate culturally held beliefs, values, and taken-for-granted practices (Leininger, 1999; Polit & Beck, 2017; Van Manen, 1984). This approach has previously been used to collect data for older adults’ lived experiences (Zhu & Zhang, 2019). By interpreting the meaning through the voices of the participants, further insights into the ways-of-being of these older adults are achieved (Kakkori, 2009).

To align with qualitative research principles, we considered the credibility, transferability, dependability, and confirmability of our research design (Lincoln & Guba, 1985). Credibility was sought through lengthy, in-depth interviews with participants (in two geographical cities), with multiple interviewers. The rich data extracted from the interviews adds credence to transferability as it infers the relevance of the participants’ voices to others in similar situations. The use of two geographical populations of older adults also adds to the transferability. Our interactive research team added a layer of reflexivity during the study which contributed to the confirmability and dependability of our data. Scrupulous records of interactions with participants were kept. Each of these efforts contributed toward the key principles of qualitative research.

### Sample and Setting

The sampling of participants occurred from two Western Canadian cities. Convenience sampling with snowball selection was used to recruit participants who (a) self-identified as having Chinese heritage; (b) were aged 70 and older; (c) lived independently in a Canadian city; and (d) made their own decisions to participate in the study. Invitational posters were placed in ethnic grocery stores, community centers, and social media platforms. A total of 11 participants were interviewed for the study. Confidentiality was assured to the participants, and pseudonyms with the prefix of “Auntie” or “Uncle” are used, as these are respectful ways to refer to older adults in Chinese culture. Research teams from each city coordinated the research process to ensure consistency in the study. Ethics approvals were obtained from two Canadian Universities.

### Data Collection

A set of interview questions was created by the research team and provided to each participant (see Table 1). The seven questions were written in both Traditional and Simplified Chinese, as well as English to ensure participants could fully grasp their meanings. Throughout all 11 interviews, a set of open-ended questions were asked, so that participants could share their thoughts and opinions freely (Aryal, 2020). Members of the research team and participants met in places of the participants’ choosing, and interviews were recorded.

**Table 1.** Interview Questions.

Interview questions
• Can you tell me a bit about yourself? • Can you please describe the meaning of relationship with relation(s) such as family members, community groups, companions and so on. • If you had a magic wand, is there anything you want to change in your life? What would it look like? • How do you get around in your everyday life (shopping, appointments, visiting friends)? • Can you please describe your typical day? What are the things that you do everyday that are important to you? Why are they important to you? How do they contribute to your quality of life? • What information would you like to have about your own health to help you live your life to the fullest? • Is there anything else that you would like to share with me?

The interviews were completed in either Mandarin or Cantonese, depending on the participants’ choosing and the questions were presented to them in advance. The interviews were transcribed into written Chinese, then translated into English for further analysis. A double translation method was used to ensure data accuracy. Without knowing the original transcript, an independent Chinese-English translator was responsible for translating all 11 interview transcripts back into Chinese. By comparing the newly translated document to the original transcription, the researchers ensured that the interviews’ content was correctly interpreted.

### Data Analysis and Validity

A systematic thematic approach guided the data collection and inductive data analysis process (Braun & Clarke, 2006 ; Nowell et al., 2017), with adherence to interpretive description methodology (Thorne, 2016). For confidentiality, all identifying information was removed from transcripts and field notes. Data analysis began after the transcription of the first few interviews. The researchers independently read and reread the transcripts, and wrote reflexive notes before developing a preliminary codebook (Thorne, 2016) in consultation with the research team. Each of the transcripts were coded and comparisons were made across the data set, seeking relationships within the data (Thorne, 2016). Through this in-depth immersion, the data was organized into categories with similar patterns. A thematic analysis flowchart was utilized to explore the themes as they were discovered. This process of analysis required continued immersion in the data and included biweekly discussions with the research team to uphold validity of the study. To conclude this process, a secondary literature review was conducted to compare the study findings with previous research. This inductive iterative approach allowed for a thorough exploration of what matters most to older adults.

Throughout the analysis process, the findings were organized and reorganized to explore the descriptions and experiences as described by the participants. A variety of steps were taken to enhance the validity of the study. A reflexive journal was maintained for the purpose of bracketing throughout the research process (Tufford & Newman, 2010). Within our team of researchers and research assistants, most of us are immigrants; furthermore, each of the team members who are of Chinese heritage speak and write two different Chinese dialects and characters, and are originally from different regions of China. The diversity of team members’ backgrounds added richness to the interpretation of the data. Ongoing contact between the researchers provided further discussion on emerging themes and categories. Field notes, usually in the form of meeting minutes, were recorded for each team meeting to assist in the analytic process (Polit & Beck, 2012). These notes often included reflections pertinent to the interviews. An audit trail contained in the recorded meeting minutes was maintained to document decisions made by the researchers, which allowed for critical thinking of the conclusions about the data. Space triangulation of data sources was accomplished by interviewing participants from two different Western Canadian cities.

The interpretive description methodology also requires a communication style designed to build rapport, and an allowance for altering initial interpretations (Thorne, 2016); each of these aspects were incorporated throughout the analysis phase. In addition, we were acutely aware of experiential knowledge, the context of different situations, and understanding that multiple realities exist (Thorne, 2016). It was our intent to accept and respect different viewpoints, and to create contexts within ethical spaces that were mindful of “different understandings and provides . . . an avenue for creating knowledge that is beneficial to communities” (Vukic et al., 2012, p. 149). These goals were at the forefront of this study.

## Findings

### Synthesis and Interpretation

A core theme and four categorical themes were identified through the data analysis. The core theme of *cultural foundations* was interwoven throughout the findings and provided the structure for the participants’ ways-of-being in the world. Four categorical themes of “it’s the little things that matter,” “making the best out of life,” “taking care of each other,” and “the need to understand” were identified as priorities in the lives of older adults in this study.

### Core Theme: Cultural Foundations

During the data analysis, a core theme of *cultural foundations* emerged. Participants identified the importance of culture and frequently referred to ways of life when they lived in China, Hong Kong, and Taiwan, and how those influences interact with their lives in Canada. The cultural foundations were evident through examples such as dietary preferences and lifestyle choices that bring comfort, family values, and general “ways of-being” with others. Being able to rely on their cultural foundations gave participants the structure for their lives that they need. This core theme of cultural foundations informs the findings of this study.

### Theme One: It’s the Little Things That Matter

*It’s the little things that matter* is an overall statement that describes the complexity of Chinese older adults integrating everyday activities into their lives in Canada. Three sub-themes are evident within this theme: the language gap in making daily life decisions, culturally congruent activities into daily routines, and self-balance of Eastern cultural attachments with Western lifestyles.

The first sub-theme of the language gap addresses the impact of English as an additional language on a sense of control and confidence in making everyday life decisions. While riding the bus, Auntie Ting shared “when the bus announces the next station, I don’t know what it’s saying . . . I can look at it, but I don’t know what it means.” Auntie Suk-Ping reinforced the language gap in health needs by stating: “the future will be difficult because I cannot say the English term for my illness . . . being an older adult here is so difficult.”

The second sub-theme of having culturally congruent activities in their daily lives shines through in participants’ preference of activities they choose to engage in and where they seek out information. Auntie Pui Shan spoke about playing mah-jong (a traditional tile game developed in China in the 19th century): “I play it every night on my laptop,” while Uncle Ming explained he learned the majority of the news through Chinese media: “by watching Chinese TV shows and radio stations, that’s how I get my information.” English speaking media are used “to learn English” (Auntie Ting), while the majority of health information is gained through “Chinese TV, Chinese newspapers, magazines, news” (Auntie Zhengqiu).

Participants spoke about the third sub-theme of seeking self-balance of Eastern cultural attachments while living a Western lifestyle. The traditional Eastern concept of filial piety is actualized differently due to the influence of Western living arrangements. Participants live in their own dwellings, away from children: “my daughters live in the States . . . they all have their own families and have to work” (Auntie Suk-Ping). Uncle Xing stated, “we don’t want to rely on them [children], so we live on our own and we do things on our own.” Auntie Yuk Lam shared “my two sons don’t give us money. My daughter gives us money, but not a lot, the money is for her dad to have tea.” Older adults sought out support from their children as needed, “places I don’t know how to get to, [my daughter] usually helps out. She accompanied me when I got my US Visa” (Auntie Ting). Auntie Oi Ling summarized “Generally speaking, we do not bother them unless it’s something special. Just something important, special, like the bank, we would call them and communicate with them. Because we cannot understand things over the phone.”

### Theme Two: Making the Best Out of Life

*Making the best out of life* refers to participants’ resilience to adapt to life changes, being open-minded, and having a positive attitude toward life, home situations, or health challenges. As life evolved, they made meaning of their circumstances and events that occurred with aging, through cultural philosophies. Auntie Pui Shan described: “because I don’t really care about life or death, I’m very open to the idea. I’m 80 years old; I might die anytime. Even if I die tomorrow, it doesn’t matter, just be open-minded.” Auntie Zivi further indicated: “God never said that life would be perfect, that’s impossible. Your life is like the ocean; there will be waves and calm times, so I have to accept the changes in life. This allows me to be more peaceful.”

**Figure 1.**
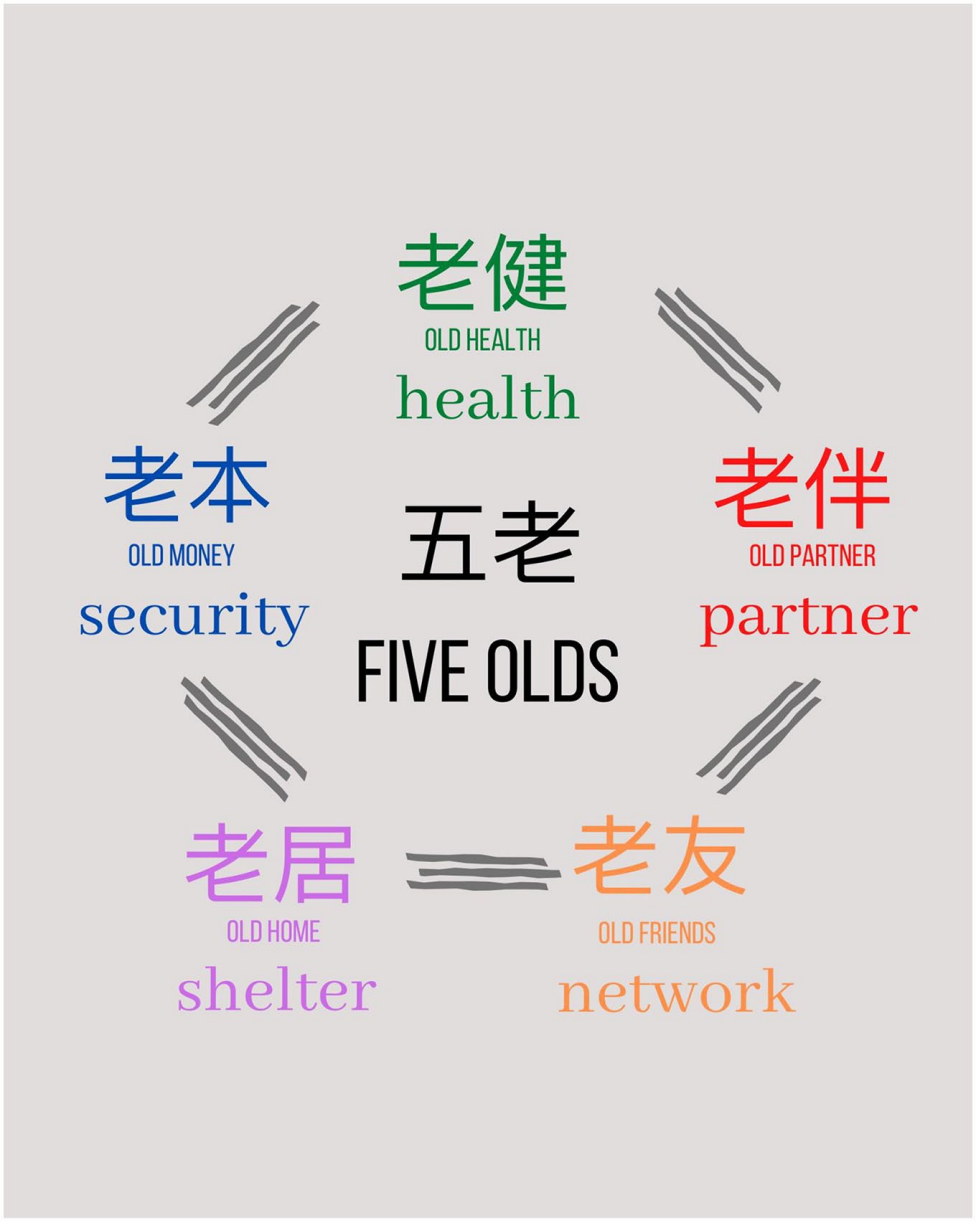
Five Olds Diagram

This theme also includes the sub-theme *The Five Olds*. Because of the participants’ resilience in making the best of their circumstances, they saw the Five Olds in their lives. The Five Olds, or Wu Lao, are a foundation for being, and refer to the importance of having money, a partner/spouse, friends, a home, and health in one’s life (see Figure 1). If older adults know that the Five Olds are there for them, then they will be okay in their lives. Uncle Xing explained:

The five olds (Wu Lao), guide how old people should live their lives . . . the first one is “old partner” (Lao Ban). So, like old people, husband and wife who can take care of each other, “old health” (Lao Jian), “Jian” as in health means that old people must have a healthy body. Then there is the “old home” (Lao Ju), which means to have a place to live . . . “old money” (Lao Ben) means to have money. And then there are “old friends” (Lao You). These are the five olds. Us old people agree these are the basic necessities (Uncle Xing).

### Theme Three: We Take Care of Each Other

*We take care of each other* is the theme that shows participants’ appreciation of relationships and social support networks; valuing family, neighbors, and friends that are new and old, near and far. Auntie Yuk Lam talked about the strength of spousal relationships,

if you are close to your parents, they cannot be with you your entire life, if you are close to your children, they will grow up and have their own family. So, the closest person to you will always be your husband

and further explained “we don’t have to eat alone. The two of us talk about our grandchildren and reflect on our old days . . . the two of us rely on ourselves.” Auntie Zivi explained how having connections with neighbors helps contribute to a sense of safety, with the use of a well-known Chinese idiom “遠親不如近鄰” (a near neighbor is better than a distant family cousin). The meaning of this idiom is that a close and trusted neighbor is of more value than relatives who live far away, and it speaks to the importance of one’s social network to offer assistance and support during times of need. In addition to neighbors, Uncle Xing shared how hard it is to make new friends after immigrating to a new country: “we know some people here, but so few of them are true friends. We think that friends are important, it’s different from family, friends can help you a lot, it’s like having another option in life, another journey.” Uncle Ming further explained the power of connecting with friends “even though we both live alone; he is only here to help me out if I need anything . . . [A]s for me, I would make some soup for him if he gets sick, we take care of each other.”

### Theme Four: The Need to Understand

*The need to understand* refers to the desire for knowledge, learning, and growth evident in the data. These desires to know appeared to be driven by culture, and provided a foundation for learning, surviving, and thriving within the Canadian landscape. Several participants referred to their involvement with community organizations, and how “Canada’s work in the community has affected me greatly . . . the government helps new immigrants, helps them with adjusting to this new environment” (Uncle Ming). Auntie Ooi Ling expressed that life was more fulfilling when

we interacted with the outside world. [We] eventually knew about organizations we could go to if we needed help. We like to know what is happening, that was when we became familiar with things . . . it was related to our interaction with society.

Auntie Ting referred to specific life skills that are needed in Canada: “everything, like how to do my taxes. I try to go to everything [seminars] . . . I want to learn everything,” and the feeling of self-fulfillment that this learning provides, “I’m proud of myself. I did a lot of things last year, very eventful in a short time period.” Auntie Ting further expressed desire “to try things that I never got to do before, dancing, cooking, and English—they all have benefits.” And finally, participants spoke about the need to hear differing viewpoints as they were learning, “everyone has different interpretations on things . . . all perspectives have their values, so I try to listen to them all” (Auntie Ting).

## Discussion

### Implications

Examining those factors relevant to what matters most to older adults is an important question to address. This qualitative study has considered this very question with a specific, yet diverse, group of Chinese Canadian older adults spread across two distinct geographic areas in Western Canada. Four themes emerged from this work:

1. It’s the little things that matter;
2. Making the best out of life (and the sub-theme of the Five Olds);
3. Take care of each other;
4. The need to understand.

Theme one speaks to the notion of cultural foundations as vital in anchoring Chinese older adults as they cope with their circumstances. Maintaining some element of their country of origin and their cultural practices offers them solace and anchors their sense of resilience to face the challenges of living in Canada. These foundations provide them with the psychological buffers to adapt to the myriad of differences between mainland China, Hong Kong, Taiwan, and Canada. Zhang et al. (2018) suggest that “personal resilience not only moderates the negative effect of adversity but also facilitates the positive effect of external resources” (p. 959).

Our initial impression of the second theme was more of an attitudinal positioning of the older adults’ resignation to, and acceptance of what they had. However, further insight into these participants is captured with an idiom expressed by Uncle Xing. The Chinese social proverb of the Five Olds (husband/partner, home, money, friends, and health) elegantly and succinctly captures some of the pivotal issues vital to these older adults’ health and wellness. According to Iakovleva and Nikolaeva (2016): “proverbs serve as the great source for a thorough insight into a culture and values that constitute its core.” The Five Olds speak to the strong hope for health, security, and relationships. These factors seem to be inextricably linked to the continued welfare of the study participants and the importance of these factors cannot be understated.

Another Chinese idiom exemplifies the third theme, “a near neighbor is better than a distant cousin.” The initial interpretation of this idiom is unidirectional, where help received from a neighbour is better than an inaccessible family member. However, a more in-depth look into this idea suggests an inherent need for connection where support is offered via one’s network of friends, and vice versa. The continued exchange forms the basis of connectedness and reflects the need for a social network. Supported by Luo and Menec (2018), Chinese older adults with adequate social networks and support were found to have more resilience to cope with life adversities and negative emotions, leading to a more content mental state of being.

In the “need to understand” theme, there is evidence that Chinese Canadian older adults have an interest in being a part of their surroundings. They also wish to linguistically understand the Canadian health care system and how to access services to maximize and maintain health. What also emerged from the data was an appreciation for the variety of health and wellness initiatives that promote interconnectedness, physical activity, mental acuity, and a general sense of well-being. There was a sense that the older adults felt that their journey was ongoing, and not static. Zhu and Zhang (2019) concurred, stating that when “settling in the new country, senior immigrants relearn skills, survival strategies, language, culture and local knowledge. They are active learners fully engaging with society” (p. 508). This theme speaks to the importance of community, and that health is inextricably intertwined with our social network.

With this insight, it is incumbent on health care system planners to consider how to support the needs of Chinese Canadian older adults in the development of their health literacy as exemplified by the Five Olds or Wu Lao. First and foremost, a key care planning principle will be to incorporate a person-centered culturally congruent approach. Second, health information needs to be available in the Chinese language and integrated into existing care systems that are frequented by Chinese older adults. An alternative plan is to have culturally congruent materials available for those who do not have access to technology. Third, having a health care system that allows them to clarify their understanding of their health situations would be important. Hence, a bidirectionality of communication, allowing for active exchange between the Chinese older adults and Canadian health care providers, may be an essential aspect that emerges from this study. Finally, an ideal situation would be the availability of health care providers fluent in a number of Chinese dialects or ready access to medical translators.

This insight is vital to impart to student nurses. During their training, formal instruction on what “matters most” to Chinese Canadian older adults will imbue in students an understanding that this population of older adults have a desire to integrate into Canadian society, but they need culturally familiar tools to assist them to advocate for their health and wellness. It is equally important for students to understand the importance of networks, relationships, and security for this population, and nursing educators need to consider how to incorporate this into their curricula and encourage students to use various media to disseminate health information.

The findings from this study link health/wellness with interconnectedness, which is “what matters most” to Chinese Canadian older adults. An exploration of how this notion impacts the health of Chinese Canadian older adults, on a larger scale, could form the basis of a future study.

### Limitations

This study has several limitations, and its findings need to be carefully interpreted. The small sample size of the study may not be representative of general Chinese older adult immigrants in Canada. The study took place in 2 of 11 provinces in Canada. The participants’ responses were not differentiated by their sociodemographic characteristics such as gender, age of immigration, level of education, place of origin (e.g., mainland China or Hong Kong), and household income. These characteristics may have significant influences on the older immigrants’ ability to adapt and adjust from traditional Chinese to Western culture. Future study could provide insights on the correlation of acculturation experiences on perceived quality of life for Chinese older adult immigrants in Canada.

## Conclusion

In summary, the results of this study suggest a need for health care providers to develop mindfulness when caring for Chinese older adults. The meaning of cultural health values and beliefs are deeply rooted and have profound impact on the outcomes of individuals’ health and healing journeys. What may seem insignificant may, in fact, make a world of difference in the quality of life for Chinese older adults. Culturally congruent nursing practice elevates positive care outcomes and enhances the quality of life of individuals, no matter where they are from.
